# Statistical Approach to Decreasing the Error Rate of Noninvasive Prenatal Aneuploid Detection caused by Maternal Copy Number Variation

**DOI:** 10.1038/srep16106

**Published:** 2015-11-04

**Authors:** Han Zhang, Yang-Yu Zhao, Jing Song, Qi-Ying Zhu, Hua Yang, Mei-Ling Zheng, Zhao-Ling Xuan, Yuan Wei, Yang Chen, Peng-Bo Yuan, Yang Yu, Da-Wei Li, Jun-Bin Liang, Ling Fan, Chong-Jian Chen, Jie Qiao

**Affiliations:** 1 Annoroad Gene Technology Co., Ltd, Beijing, China; 2 Department of Gynecology and Obstetrics, Peking University Third Hospital, Beijing, China; 3 Department of Obstetrics, Beijing Obsterics and Gynecology Hospital, Capital Medical University, Beijing, China; 4 The Obstetric Department of First Affiliated Hospital of Xinjiang Medical University, Urumqi, China; 5 Department of Obstetrics and Gynecology, Beijing Friendship Hospital, Capital Medical University, Beijing, China; 6 Department of Obstetrics and Gynecology, First Hospital of Shanxi Medical University, Shanxi, China

## Abstract

Analyses of cell-free fetal DNA (cff-DNA) from maternal plasma using massively parallel sequencing enable the noninvasive detection of feto-placental chromosome aneuploidy; this technique has been widely used in clinics worldwide. Noninvasive prenatal tests (NIPT) based on cff-DNA have achieved very high accuracy; however, they suffer from maternal copy-number variations (CNV) that may cause false positives and false negatives. In this study, we developed an algorithm to exclude the effect of maternal CNV and refined the Z-score that is used to determine fetal aneuploidy. The simulation results showed that the algorithm is robust against variations of fetal concentration and maternal CNV size. We also introduced a method based on the discrepancy between feto-placental concentrations to help reduce the false-positive ratio. A total of 6615 pregnant women were enrolled in a prospective study to validate the accuracy of our method. All 106 fetuses with T21, 20 with T18, and three with T13 were tested using our method, with sensitivity of 100% and specificity of 99.97%. In the results, two cases with maternal duplications in chromosome 21, which were falsely predicted as T21 by the previous NIPT method, were correctly classified as normal by our algorithm, which demonstrated the effectiveness of our approach.

Cell-free fetal DNA (cff-DNA) in the maternal plasma, discovered by Lo^1^ nearly two decades ago, enables noninvasive prenatal testing (NIPT). Compared with the conventional prenatal diagnostic methods amniocentesis and chorionic villus sampling, which carry procedure-related risks of miscarriage of 0.11% and 0.22%, respectively^2^, NIPT confers no risk of spontaneous abortion and cff-DNA can be detected as early as 4 gestational weeks^3^. To date, diverse approaches have been developed for the noninvasive detection of feto-placental anomalies and structural variations, or to obtain genetic information, such as detection of chromosome aneuploidy (offered to women with high-risk pregnancies)^4^,^5^, copy number variation (CNV)^6^,^7^, whole genome measurement^8^, prenatal paternity testing^9^, monogenic disorder detection^10^,^11^, and so on.

Among the NIPT applications, the massively parallel sequencing (MPS)-based methods for feto-placental chromosome aneuploidy detection (FCAD) are currently considered suitable^12^, and they are the most widely used methods for clinical application. The stability of algorithms based on read counts has been proved many times in large-scale studies to detect feto-placental chromosomal aneuploidy with a very low false-positive rate (FPR) and false-negative rate (FNR)^13^,^14^,^15^,^16^,^17^,^18^,^19^,^20^. However, the error rate for FCAD using MPS can be decreased further.

Discordant MPS results can be attributed to several causes, such as confined placental mosaicism^21^,^22^, maternal CNV^23^,^24^,^25^, maternal mosaicism^25^,^26^, vanishing twin^27^, maternal malignancy, laboratory error and sample labeling error; among these, maternal CNV plays a crucial role in calculation of the Z-score that is used to analyze the result. Maternal duplication boosts the number of relative unique mapped chromosome reads, increases chromosomal coverage, and results in a higher Z-score than the normal standard, intensifying the risk of false-positive results. Maternal deletion influences the result in the opposite way, increasing the risk of false negatives. Study of MPS-based prenatal screening has confirmed that some false-positive results are caused by maternal CNV^23^,^24^,^25^.

Great progress has been made in resolving the limitation in read count statistics by separating chromosomes into bins, to calculate a much more robust statistic for FCAD, and thus revising chromosome coverage^28^. In recent years, some studies have succeeded in using a binned approach to avoid the misinterpretation caused by maternal CNV^25^,^29^,^30^. Within-sample reference bins and the sliding window Z-score method allow WISECONDOR to obtain a fixed Z-score, regardless of some aberrant bins with significantly high or low Z-scores caused by small maternal CNV^29^. A four-parameter integrated pipeline proposed by Bayindir uses the median of the Z-scores measured per bin in the chromosome to gain a robust Z-score, despite the occurrence of some small maternal CNV^30^. FCAPS divides the human genome into 99%-overlapping sliding bins, and utilizes a binary segmentation algorithm to compute CNV breakpoints. The supposed maternal CNV segments with a very low or high t-score will be removed from the basic analysis in order to obtain an unaffected t-score^25^.

The latest research has shed light on the possibilities of a NIPT screening test for trisomy 21 (T21), trisomy 18 (T18) and trisomy 13 (T13)^23^,^31^,^32^. However, NIPT screening tests suffer from low positive predictive values and high “no call” rates, even though the FPRs are lower than for standard screening tests. Biological phenomena such as maternal CNV are held accountable for some discrepant results. A study reported by Snyder^24^ described the effect of maternal CNVs on FPRs, and suggested continued investigation and refinement of methodological approaches for FCAD.

We were therefore motivated to develop a stable shot-gun MPS-based NIPT FCAD workflow for T21, T18 and T13, avoiding the maternal CNV effect, based on the mathematical correlation between maternal CNV and chromosomal coverage. In the study, an algorithm called MAT-CNV was used to detect maternal CNV and eliminate its influence on chromosome coverage. In addition, a method based on feto-placental concentration difference (FCD) was used to help decrease FPR. At the end of this paper, we discuss the limitations of MAT-CNV and the circumstances under which FCD could achieve better outcomes.

## Results

### NIPT FCAD workflow

Supplemental Figure 1 conveys the workflow followed with the 6615 pregnant women. Cell-free DNA was extracted and 5–10 million short reads were generated for each enrolled sample. Reads that uniquely mapped to the human genome were retained. Unique mapped reads in each 100 kb window bin in the chromosomes were counted and adjusted by GC bias in each window using LOWESS. The read counts in window bins in each chromosome were summed to compute chromosome coverage. Simultaneously, read counts in window bins were used to detect maternal CNVs. If maternal CNVs > 300 kb in length were found, chromosome coverage was calibrated to eliminate the maternal CNV effect by utilizing a refinement function described in the Method. A Z-score normalization was applied to detect fetal aneuploidy, using the adjusted chromosomal coverage. Fetal aneuploidy was defined by an absolute Z-score above 3. *Z*_*fetal*_, representing the degree of difference between the two fetal concentrations, inferred from chromosome X and the aneuploidy chromosome, was computed to filter out false positives further. A result was considered as false positive if *Z*_*fetal*_ > = 3.

### Simulation Result

Six groups of simulated maternal plasma data for chromosomes 13, 18, and 21 were generated, based on Poisson distributions, to validate the MAT-CNV approach, under the assumptions that maternal duplication/deletion was not inherited by the fetus. Each group consisted of different maternal CNV sizes (0.5–5 Mb) in 0.25 Mb steps and dissimilar fetal concentrations (5%, 10%, and 15%).

We first evaluated whether α, the key parameter in our adjustment model, as detailed in the Methods, exactly represents the maternal CNV effect. The real maternal CNV effect value was computed as the chromosome coverage without any maternal CNV divided by the simulated chromosome coverage with maternal CNV in different circumstances. As shown in Supplemental Figure 2 and Supplemental Figure 3, the parameter α and real maternal CNV effect fitted the linear model *y* = *x*. The deviation of the two values was measured by the Shapiro–Wilk test. In most cases, the P-values were above 0.05, so we could not reject the null hypothesis that α equals the real maternal CNV effect value, implying the theoretical achievement of the desired outcome of our adjustment approach.

**Figure 1:**
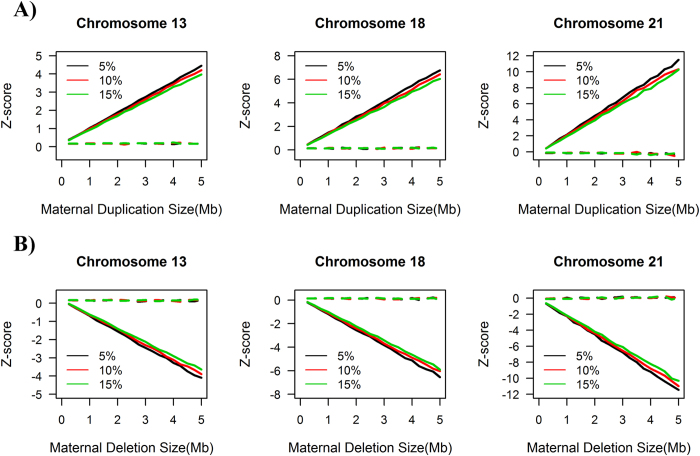
Simulation results for maternal duplication (**A**) and maternal deletion (**B**) in chromosomes 13, 18 and 21. olid lines represent raw Z-scores of the simulation result and dashed lines indicate Z-scores after our maternal CNV (MAT-CNV) adjustment. The black, red and green lines represent fetal concentrations of 5%, 10% and 15% respectively. The x-axis indicates the size of maternal CNV, while the y-axis shows the Z-score.

We subsequently investigated the effect of maternal CNVs on the final Z-score calculation. As demonstrated in Fig. 1, the raw Z-scores, before revision by our MAT-CNV approach, increased or decreased in proportion with the size of the maternal duplication or deletion respectively, although the degree of correlation varied in terms of fetal concentrations and different chromosomes. Higher fetal DNA fraction and longer valid chromosome length reduced the influence of maternal CNV on the raw Z-score. Apparently, a maternal duplication of more than 1.5 Mb on chromosome 21, whatever the fetal concentration, resulted in a Z-score larger than 3 in a euploid fetus, leading to a false-positive NIPT result. The thresholds of maternal CNV size that will cause a false-positive NIPT result in chromosomes 18 and 13 are 2.2 Mb and 3.2 Mb, respectively. Remarkably, after applying the MAT-CNV approach, the Z-scores remained around 0 under all circumstances, implying that our approach was successful in reducing discrepant NIPT results caused by maternal CNVs.

### Pregnant Women

**Figure 2:**
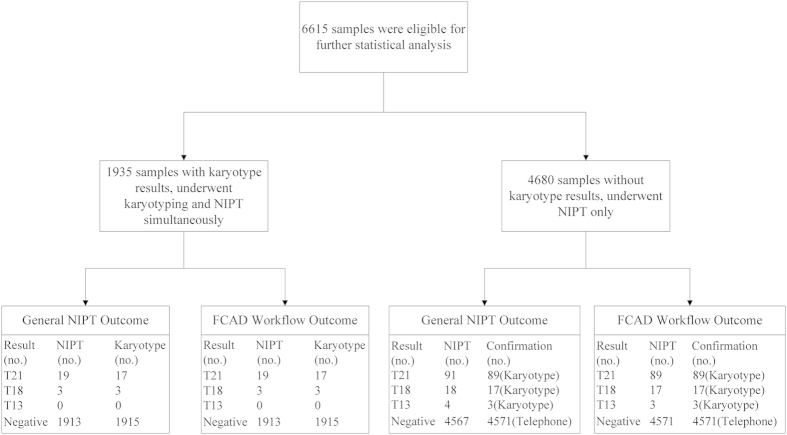
Performance of the general NIPT method and the FCAD workflow for 6615 patients.

A total of 6615 pregnant women were recruited, 1935 of whom underwent the standard karyotyping test and NIPT FCAD test simultaneously. The remaining 4680 first underwent NIPT FCAD; these results were later confirmed by either karyotyping or follow-up visits (Fig. 2). The average age of the patients was 32.7 years (standard deviation (SD) = 5.3), and 47.5% of patients were >35 years of age. In total, 98.37% of the patients were in the second trimester and the average gestation week was 19 (SD = 2.6).

### Clinical Outcomes

We compared the results of the NIPT FCAD workflow with a simple version of this workflow called the “General” NIPT, which lacks MAT-CNV and FCD refinement (Fig. 2). In 1935 samples that underwent both the NIPT test and the karyotyping test, both NIPT methods detected 19 cases of trisomy 21, of which 17 were confirmed by karyotyping, and three cases of trisomy 18, all confirmed by karyotyping. In 4680 samples which only underwent NIPT testing, our NIPT FCAD workflow detected 89 cases of trisomy 21, 17 cases of trisomy 18 and three cases of trisomy 13, all of which were later confirmed by karyotyping. Negative results from the NIPT test were confirmed by telephone follow-up. Compared with the results from our workflow, the general NIPT method generated four false positives: two for trisomy 21, and one each for trisomy 18 and trisomy 13. When compared with previous general NIPT outcomes, our innovative approach with the adjusted workflow increased the accuracy ratio by 0.03% from 99.94% to 99.97%, 0.02% from 99.98% to 100%, and 0.02% from 99.98% to 100% for chromosomes 21, 18 and 13, respectively (Table 1).

### MAT-CNV Adjustment

Among the NIPT FCAD results, we applied our MAT-CNV adjustment approach to find two patients who had small maternal duplications in chromosome 21; the NIPT results of these two samples were altered from T21 to normal.

**Figure 3:**
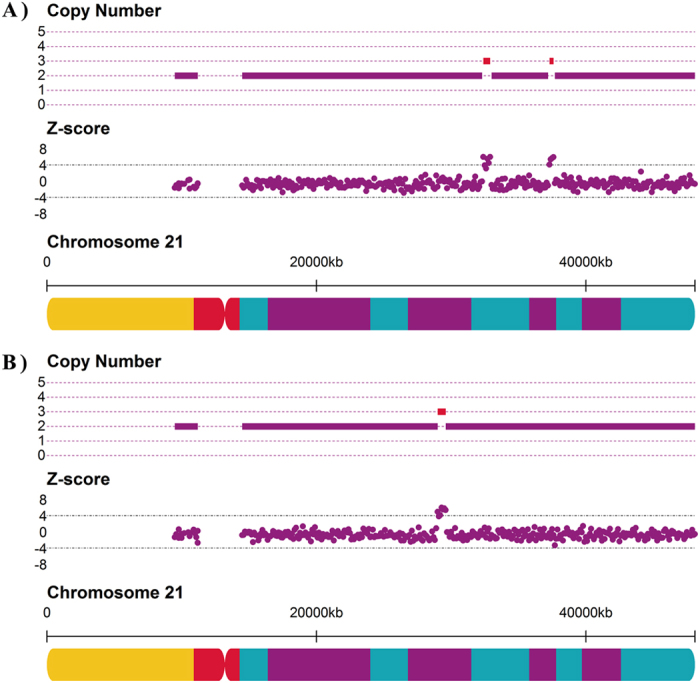
Detection of maternal copy number variations (CNVs) in samples EK01875 (**A**) and BD01462 (**B**). **A**) in sample EK01875, two duplications with a copy number of 3 were found by our MAT-CNV procedure in 21q22.11 and 21q22.12. (**B**) in sample BD01462, a 700 kb duplication with a copy number of 3 in 21q21.3 was found. Red lines indicate the CNV regions.

**Figure 4:**
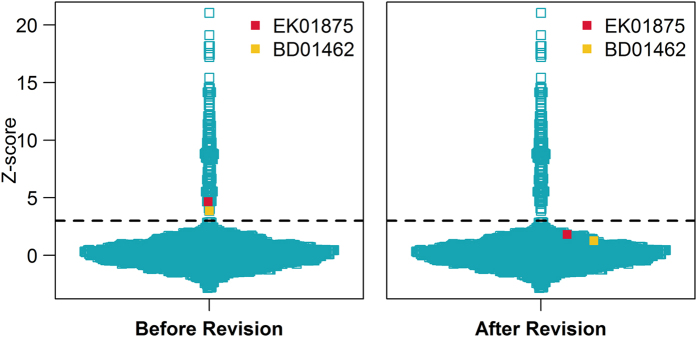
Z-scores of chromosome 21 before and after the noninvasive prenatal test FCAD adjustment workflow. The orange and red squares indicate samples BD01462 and EK01875, both with specific CNVs in chromosome 21, and the blue squares represent the other 6613 samples. The dashed lines refer to the Z-score threshold of 3. The y-axis indicates the Z-score.

The first case involved a 36-year-old pregnant woman (EK01875). The NIPT FCAD test was performed at 18 gestational weeks. In the maternal CNV detection process, we identified two segmental duplications (×3) in chromosome 21, that is, ~500 kb at 21q22.11 (32,361,194–32,861,193) and ~350 kb at 21q22.12 (37,261,194–37,611,193) (Fig. 3A). To confirm these findings, genomic DNA from maternal white blood cells was interrogated using an SNP-array (Affymetrix CytoScan 750k Array, BEIKANG Inc., Beijing, China). The two CNVs were also detectable in the array results, as shown in Supplemental Figure 4, although the aggregated size (~750 kb) was slightly smaller. The parameter α of chromosome 21 in this sample was 1.012, which resulted in the revision of the Z-score of chromosome 21 from 4.66 to 2.36, thus changing the NIPT conclusion from trisomy 21 to normal diploidy (Fig. 4). The patient also decided to undertake a standard karyotyping test at 21 gestational weeks, which confirmed the diploid karyotype of the fetus.

Another case involved a young pregnant woman aged 24 years (BD01462), who underwent the NIPT FCAD test at 23 gestational weeks. A ~700 kb duplication (×3) at 21q23.1 (28,911,194–29,611,193) was identified as maternal CNV by our approach (Fig. 3B). The CNV was confirmed by SNP-array with a smaller size (568 kb) and extra copy (×4) (See Supplemental Figure 5). The parameter α of chromosome 21 was computed as 1.009, which helped calibrate the Z-score of chromosome 21 from 3.87 to 1.83. Hence, our NIPT FCAD test returned a negative result, with no indication of trisomy 21. Karyotyping of the fetus, undertaken at 27 gestational weeks, also indicated a normal diploid karyotype.

### FCD Adjustment Result

**Figure 5:**
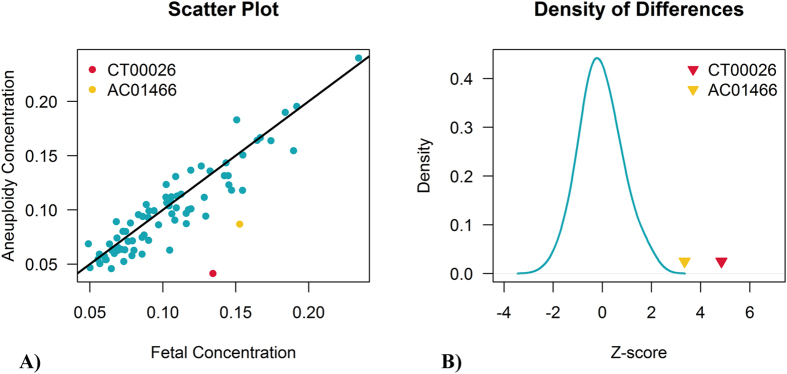
Scatter plot of two fetal concentrations (**A**) and the density of their differences (**B**). **A**) scatter plot showing the differences of two fetal concentrations. The x-axis represents fetal concentrations calculated from chromosome X; the y-axis shows fetal DNA fractions estimated from their aneuploid chromosomes. (**B**) density of the differences between two fetal concentrations. The blue solid line shows the difference distribution of positive samples. The false-positive samples CT00026 and AC01466 are colored in red and yellow respectively.

We found two cases (CT00026 and AC01466) that were classified as false-positive samples according to the FCD results after measuring the differences between two fetal DNA fractions computed from chromosome X and the aneuploid chromosome. Mathematically, two fetal fractions will be better fitted by the linear model *y* = *x*, and the difference between the two fetal DNA fractions originates from a normal distribution, which was evident in the true positive trisomy results (Fig. 5). CT00026 was a patient who underwent the NIPT FCAD test at 22 gestational weeks. The Z-score of chromosome 18 was 4.39, indicating a potential trisomy 18; however, the fetal fraction of 12.2% from chromosome X was dramatically different from the 3.5% computed from chromosome 18. This huge contrast resulted in a *Z*_*fetal*_ of 4.84 and led this patient to be considered as having a potential false-positive sample.

Another patient, AC01466, had a NIPT FCAD test carried out at 22 gestational weeks that returned a Z-score of 6.12, estimated from chromosome 13. The result was also defined as a potential false positive owing to the high *Z*_*fetal*_ of 3.35, which was computed using the fetal fraction of 14% from chromosome X and 7.7% from chromosome 13. Further karyotyping results from the two samples demonstrated the FCD determinations.

## Discussion

Our NIPT FCAD adjustment workflow achieves better results than the “General” NIPT method by decreasing FPR. Two out of two false-positive samples caused by maternal CNV were altered to negative results through our workflow, and all three CNV areas found by MAT-CNV completely supported the SNP array outcomes. Moreover, another two samples, determined as potential false positives by FCD on account of their significant deviation of fetal concentration difference (Fig. 5), were ascertained by karyotyping to show diploidy.

We have demonstrated an appealing result of MAT-CNV in decreasing the error rate of the NIPT test in our clinical results; the accurate detection of maternal CNVs is essential for MAT-CNV. The application of MAT-CNV will not lessen but rather will intensify the FPR or FNR of NIPT if the predicted maternal duplications or deletions turn out to be wrong. An inaccurate detection of maternal duplication will lead to a lower Z-score than the cut-off, and this increases the FNR of NIPT. On the contrary, incorrect detection of maternal deletions may contribute to an increase in the FPR of NIPT where Z-scores are adjusted to above the threshold. Therefore, the coefficient SD of DNAcopy, which controls the sensitivity of detection of CNV, needed to be adjusted from 3 to 4 to detect maternal CNV with confidence.

Using a strategy of highly overlapped bins and deep sequencing can contribute to increasing the accuracy of detection of maternal CNV. A more comprehensive statistical approach is required to detect areas of maternal CNV of less than 100 kb with accuracy in samples with the current limited number of mapped reads.

Another important point regarding MAT-CNV is the inheritance of maternal CNVs, because the estimation of the maternal CNV effect α is completely different under the two different assumptions. Maternal CNV apparently contributes all of the excessive or discounted unique mapped reads when the CNV is not inherited by the fetus, while the effect of maternal CNV on chromosome coverage decreases when fetal concentration increases. The difference between **Function 2** and **Function 1** is depicted as 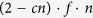, indicating that a higher α will result in a much greater decrease of chromosome coverage if maternal duplication exists; however, a lower α can help increase the chromosome coverage in cases of maternal deletion. Therefore, to avoid false-negative results, **Function 2** is used for estimating α when maternal duplication exists, while **Function 1** is used to calculate α in cases of maternal deletion.

In the study, two potential false positives were correctly detected by computing the Z_fetal_, which represents the deviation of different fetal fractions. The accuracy of the calculation of Z_fetal_ is mainly dependent on the assumption that there is no mosaicism in the aneuploid chromosome of the fetus. The fetus of case CT00026 will be close to a karyotype of (46,XY[71.3%]/47,XY,+18[28.7%]) if mosaic T18 exists (the mosaic ratio can be calculated as the fetal concentration of aneuploid chromosome divided by the fetal concentration of chromosome Y or chromosome X). The fetus of AC01466 will have a karyotype of (46,XY[45%]/47,XY,+13[55%]) if mosaic T13 exists. Therefore, samples that have a high Z-score and absolute Z_fetal_ > = 3 should also be recommended to undergo standard karyotyping when the new technique is used in routine analysis.

A low-cost sequencing epoch is approaching, and a higher number of unique mapped reads per sample can be expected. In this paper, we have presented a correlation between maternal CNV and chromosomal coverage. The simulation results and MAT-CNV based on the function showed appealing promising outcome. Although no false-negative results were detected by MAT-CNV in the clinical trial, its ability to decrease the FNR is tangible. We hope that the functions described in MAT-CNV will help other researchers to study more intricate relationships between maternal CNV and discrepant NIPT results. Another adjustment, FCD, depends on a strict assumption, which means that it is not recommended for use in routine clinical analysis.

## Methods

### Sample and Experiment

The study was approved by Peking University Third Hospital, Beijing Obstetrics and Gynecology Hospital, and Annoroad Gene Technology Clinical Laboratory (Yizhuang, Beijing). Written informed consent was obtained from all patients. In total, 6615 pregnant women were enrolled. Among the patients, 1935, who were predicted to be at high risk for aneuploidy according to high maternal age (>=35 years old), positive serum marker screening, or abnormal fetal ultrasound results, concurrently underwent standard karyotyping analysis (Peking University Third Hospital or Beijing Obstetrics and Gynecology Hospital) and NIPT FCAD testing. Karyotyping results were provided after the NIPT FCAD test as a blind study. The remaining 4680 patients first underwent NIPT FCAD testing; they were recommended to undergo karyotyping analysis if identified with positive results. Negative results on the NIPT FCAD test were validated by telephone follow-up after birth of the baby.

At enrollment, study personnel obtained a 10 mL peripheral venous blood sample from each patient. The samples were preserved and delivered in EDTA/STRECK tubes after 2 rounds of centrifugation to separate the plasma had been performed in local laboratories where the patients were enrolled. All tubes were delivered to Annoroad Gene Technology Clinical Laboratory. Cell-free DNA was extracted from plasma and underwent Illumina Hiseq 2000/2500 sequencing; 5–10 million 35 bp-length reads were generated for each sample for further statistical analysis. All experiments were performed in accordance with relevant regulations and details.

### Maternal CNV Analysis

#### Maternal CNV detection

To detect maternal CNV, chromosomes were divided into 100-kb window bins prior to obtaining the count statistics; adjacent window bins shared a 50% area of overlap. Similar to various Readdepth-based algorithms^33^,^34^, the number of unique mapped reads in each window bin was counted, adjusted in terms of GC bias and mapability ratio, and then converted to a window bin Z-score after standardization. The DNAcopy package in R was used to segment the copy number data using Z-scores to detect regions with abnormal copy number^35^

#### Correlations among maternal CNV, chromosome coverage, copy number and fetal concentration

In contrast to the usual method of computing chromosome coverage, we developed an adjustment method for calibration of chromosome coverage, to eliminate the effect of maternal CNV. The valid chromosome length, fetal concentration, maternal CNV size, copy number, and inheritance of maternal CNV were considered in this approach. Coefficient α, depicted as 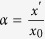, was defined to measure the effect of maternal CNV on chromosome coverage, where 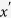 represents chromosome coverage when maternal CNV exists and 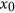 stands for chromosome coverage when maternal CNV does not exist.

When the maternal CNV is inherited by the fetus, α can be computed using the following **Function 1**,


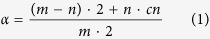


where *m* measures valid chromosome length (which can be inferred as the whole chromosome length minus the vacant area length, where vacant area means the area that unique mapped reads cannot cover, the centromeres in particular), *n* and *cn* stand for maternal CNV size and copy number, respectively. Both *m* and *n* are measured in Mb. Assuming that 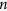 is 0 or *cn* is 2, which means there is no CNV in this chromosome, 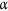 will be 1, indicating no amplification or minimization of chromosome coverage caused by the maternal CNV.

When the CNV is not inherited, α can be calculated as below. In **Function 2**, 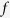 refers to the fetal concentration.


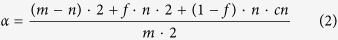


Obviously, α will be more than or less than 1 if maternal duplication or deletion was found, respectively. Chromosome coverage after removal of the maternal CNV effect is adjusted by 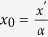. Methods of coverage normalization and fetal concentration estimation have been described previously^4^,^16^,^36^. Z_aneu_, based on revised coverage, reveals the actual Z-score without the influence of maternal CNV.

### Estimation of Fetal DNA Fraction

Fetal concentration can be estimated from the final sequence read distribution^28^. Given that chromosome X is under-represented in cell-free DNA if the fetus is male, the fetal DNA fraction can be calculated as 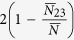, where 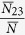 is the average number of sequence reads per bin for chromosome X normalized to the global average. From another aspect, the fetal DNA fraction could also be computed as 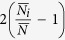, where 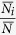 is the normalized average number of sequence reads per bin for chromosome 13, 18 or 21 for the aneuploid samples.

### Fetal Concentration Difference

For samples with positive indication of trisomy and a male fetus, two different fetal concentrations are available to evaluate fetal concentration differences. The idea of FCD is based on Hudecova’s work^37^, in which a discrepancy between the Z-score and fetal concentration was reported. Mathematically, fetal concentrations, estimated from the aneuploid chromosome and chromosome Y, will be better fitted by the linear model 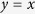 if there is no mosaicism in the aneuploid chromosome, and the difference between the two fetal DNA fractions originates from a normal distribution. 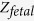, described as 
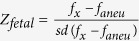
, measures the deviations of each positive sample, where 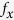 represents the fetal concentration estimated by chromosome X, 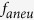 stands for the fetal DNA fraction calculated from the aneuploidy chromosome, and *sd* is the standard deviation. An absolute value of 3 is set as the 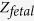 threshold. Any positive sample with an absolute 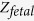 > = 3 is considered a false-positive sample.

## Additional Information

**How to cite this article**: Zhang, H. *et al.* Statistical Approach to Decreasing the Error Rate of Noninvasive Prenatal Aneuploid Detection caused by Maternal Copy Number Variation. *Sci. Rep.* **5**, 16106; doi: 10.1038/srep16106 (2015).

## Supplementary Material

Supplementary Information

## Figures and Tables

**Table 1:** Statistics of the general noninvasive prenatal test (NIPT) method and our workflow results.

	General NIPT			Our Workflow		
	Chr21	Chr18	Chr13	Chr21	Chr18	Chr13
Accuracy Ratio	99.94%	99.98%	99.98%	99.97%	100%	100%
False Positive Ratio	0.06%	0.02%	0.02%	0.03%	0%	0%
False Negative Ratio	0%	0%	0%	0%	0%	0%
Positive Predictive Value	96.36%	95.24%	75%	98.15%	100%	100%
Negative Predictive Value	100%	100%	100%	100%	100%	100%
