# Supplementary material for: Statistical Approach to Decreasing the Error Rate of Noninvasive Prenatal Aneuploid Detection caused by Maternal Copy Number Variation
Source: Sci Rep. 2015 Nov 4;5:16106. doi: 10.1038/srep16106 (PMC4632076; doi:10.1038/srep16106)
Supplement: Supplementary Information [file srep16106-s1.pdf]

# Statistical Approach on Decreasing Error Rate of Noninvasive Prenatal Aneuploid Detection caused by Maternal Copy Number Variation

Han Zhang[1+], Yang-Yu Zhao[2+], Jing Song[3+], Qi-Ying Zhu[4+], Hua Yang[5+], Mei-Ling Zheng[6], Zhao-Ling Xuan[1], Yuan Wei[2], Yang Chen[1], Peng-Bo Yuan[2], Yang Yu[2], Da-Wei Li[1], Jun-Bin Liang[1], Ling Fan[3*], Chong-Jian Chen[1*], Jie Qiao[2*]

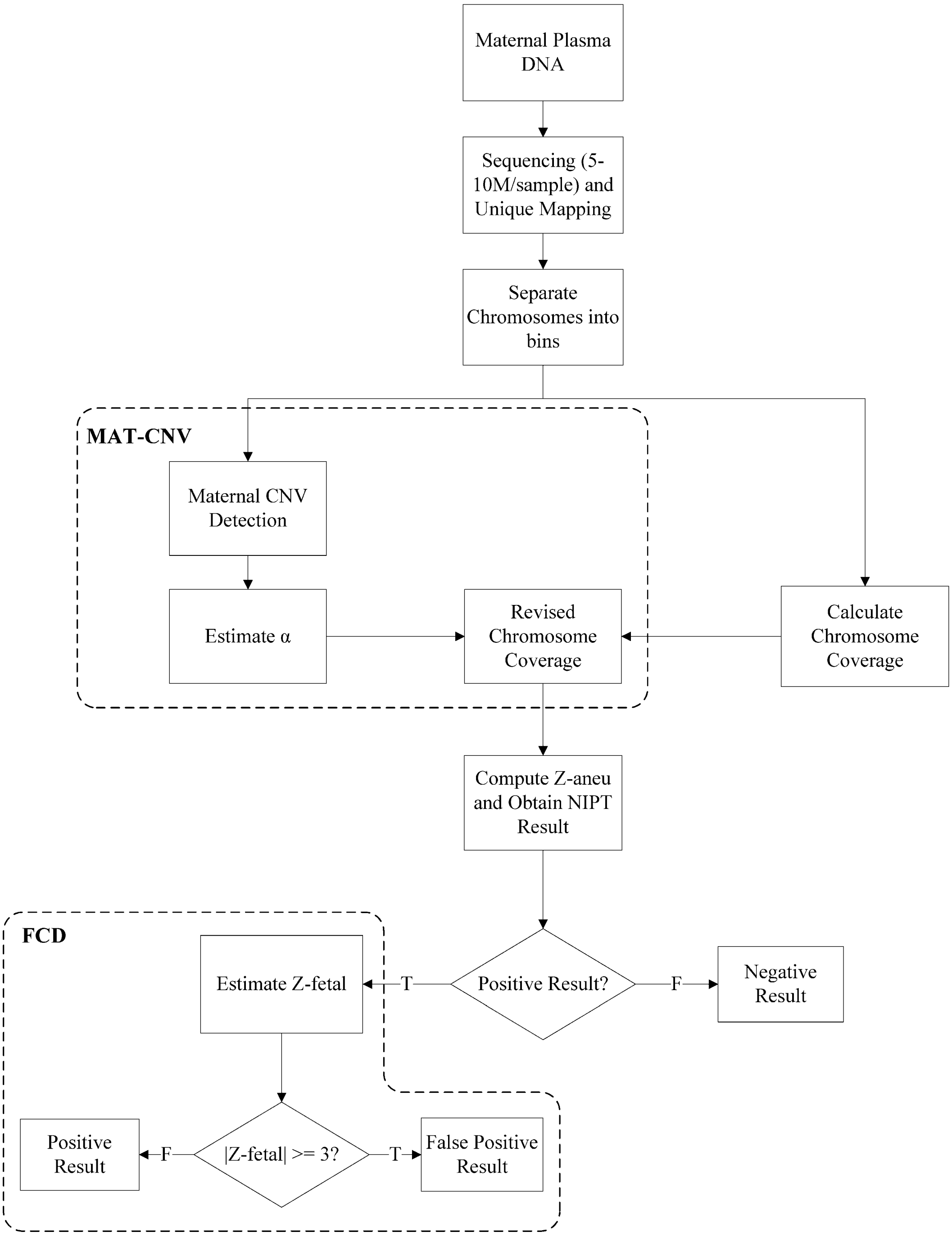


Supplementary Figure 1. The NIPT FCAD workflow.

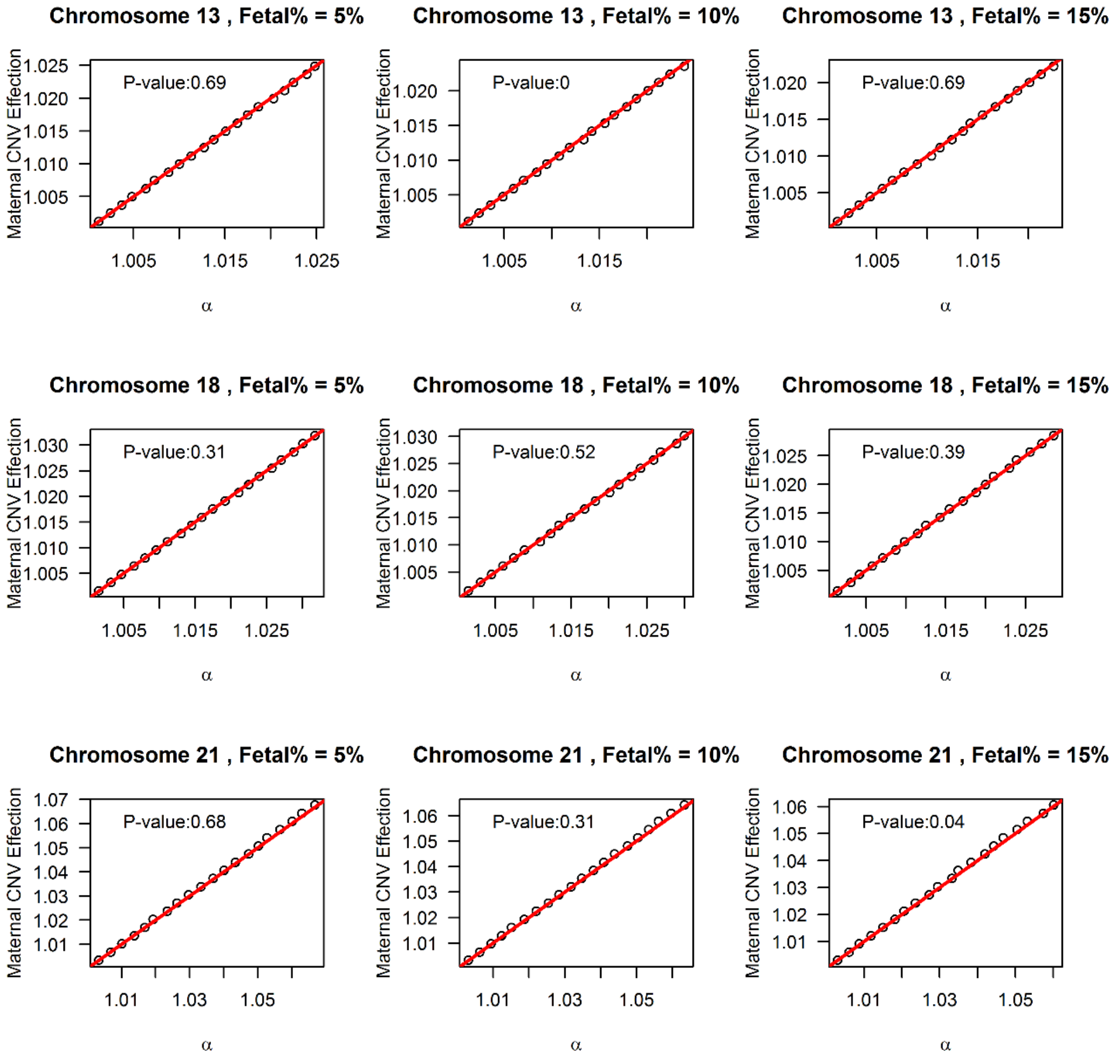


Supplementary Figure 2. Evaluation of the parameter α in the presence of maternal duplication. The relationships between the parameter α and the real maternal CNV effect are plotted for chromosomes 13, 18 and 21 in the case of fetal concentrations of 5%, 10% and 15%, respectively. Red lines represent the function $y = x$. The P-value in each plot was computed using the Shapiro–Wilk test.

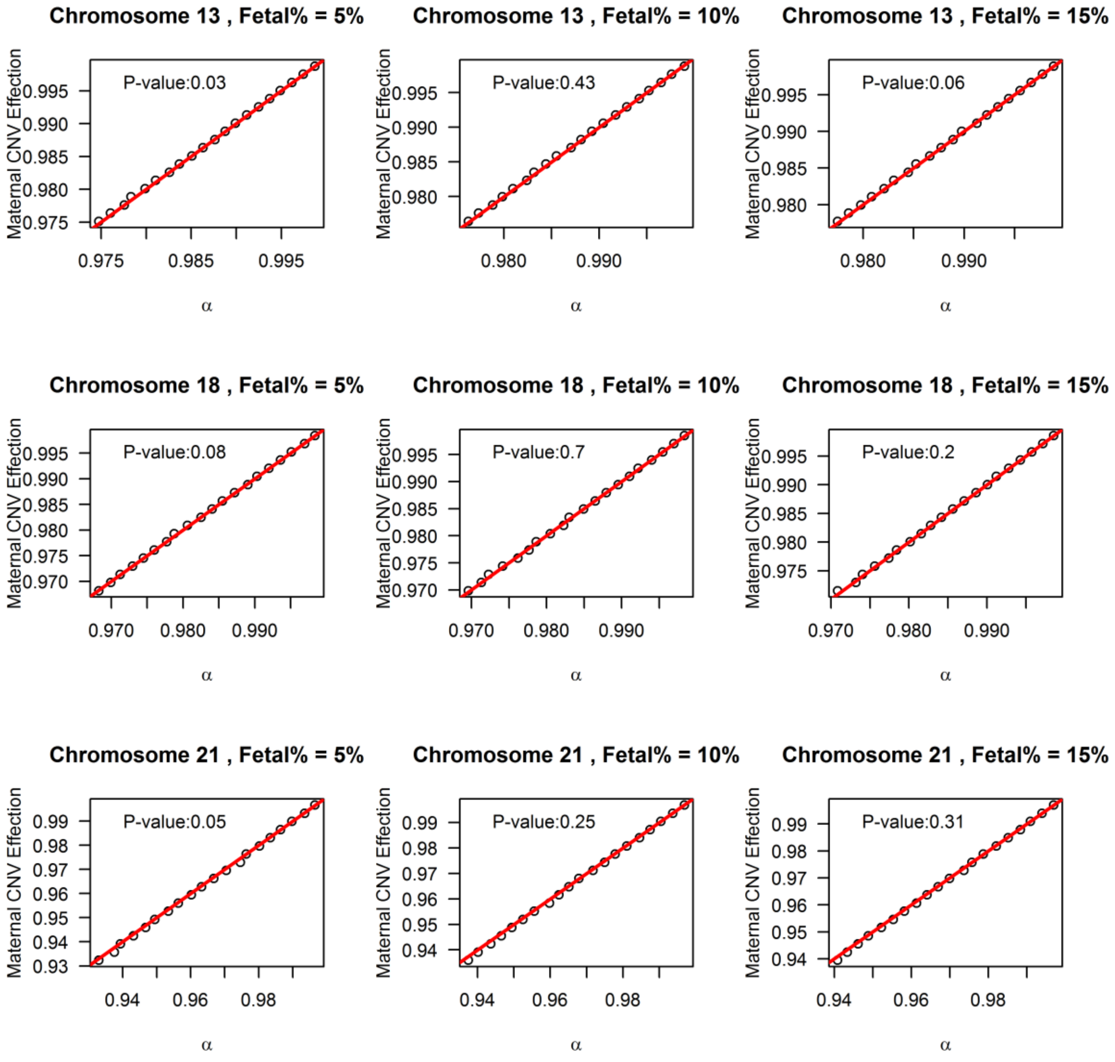


Supplementary Figure 3. Evaluation of the parameter α in the presence of maternal deletion. The relationships between the parameter α and the real maternal CNV effect are plotted for chromosomes 13, 18 and 21 in the case of fetal concentrations of 5%, 10% and 15%, respectively. Red lines represent the function $y = x$. The P-value in each plot was computed using the Shapiro–Wilk test.

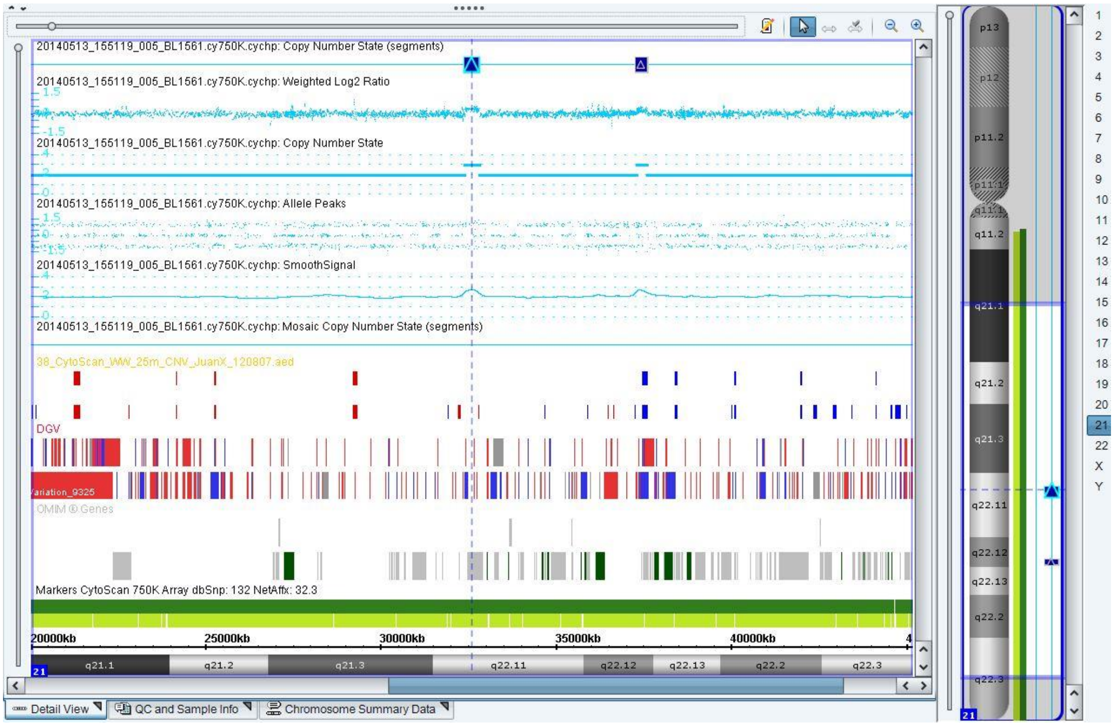


Supplementary Figure 4. Confirmation of maternal duplication in chromosome 21 in EK01875 using SNP-array.

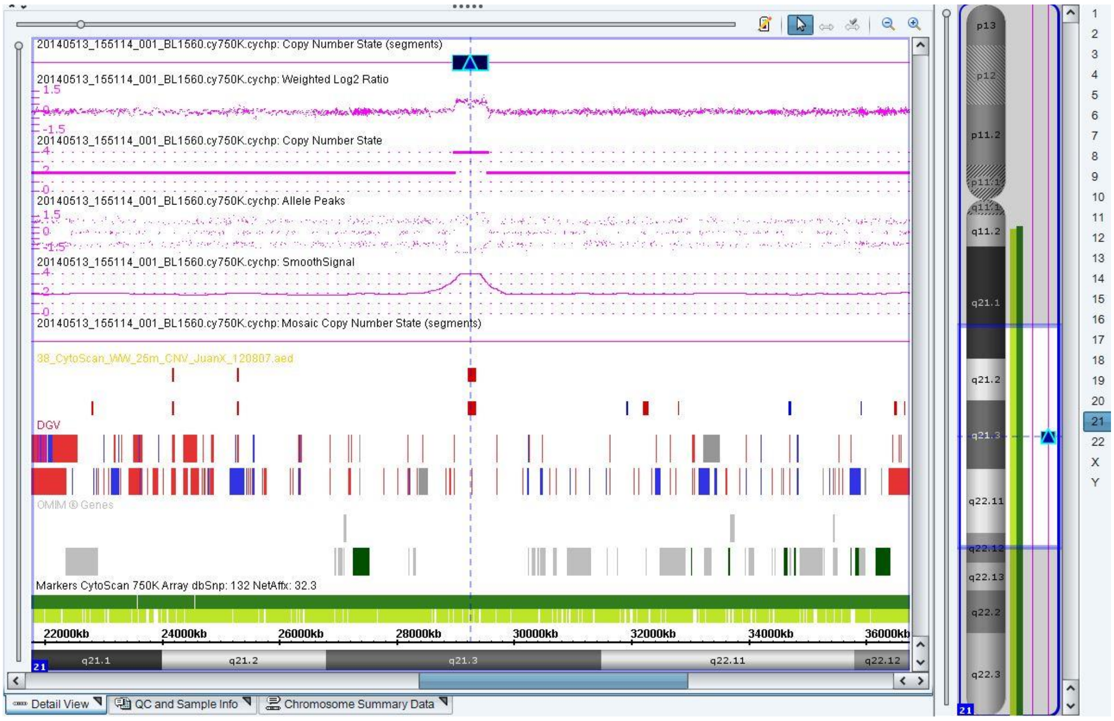


Supplementary Figure 5. Confirmation of maternal duplication in chromosome 21 in BD01462 using SNP-array.
